# Effectiveness and efficiency of Virtual Reality cognitive behavioural therapy for paranoid delusions - results from a randomized clinical trial

**DOI:** 10.1192/j.eurpsy.2025.54

**Published:** 2025-08-26

**Authors:** W. Veling

**Affiliations:** Department of Psychiatry, University Medical Center Groningen, Groningen, Netherlands

## Abstract

**Abstract: Background:**

Cognitive behaviour therapy is the main evidence-based psychological treatment for paranoid ideations in patients with psychotic disorders. Virtual reality may improve psychological treatment, because it facilitates behaviour interventions aimed at reducing avoidance and dropping safety behaviours. We investigated the effects of virtual-reality-based cognitive behaviour therapy for paranoid ideations (VR-CBTp) compared to standard CBTp.

**Methods:**

We performed a pragmatic single-blind, randomised clinical trial in seven mental health centres in the Netherlands and Belgium. Eligible patients had a psychotic spectrum disorder and experienced paranoid ideations. Both interventions consisted of 16 sessions maximum. Treatment could be completed early when all goals had been achieved. The primary outcome was momentary paranoia, measured with the experience sampling method (ESM). Secondary outcomes included other measures of paranoid ideations, safety behaviour, social anxiety, depression, worry and self-esteem.

**Findings:**

103 participants were enrolled and 98 randomised to VR-CBTp (n=48) or CBTp (n=50). At post-treatment, VR-CBTp had significantly stronger effects than standard CBTp at post-treatment on momentary paranoia (interaction effect b=-0·3, 95% CI -8·4 to 7·8, n=81, p=0·04, effect size 0·62), safety behaviour, depressive symptoms and self-esteem, of which the difference in effects on self-esteem and social interaction anxiety remained at follow-up. Completers on average received 12·4 (VR-CBTp) and 15·0 (CBTp) sessions. Limited ESM compliance resulted in 43% data loss at post-treatment and 49% at follow-up.

**Interpretation:**

CBTp and VR-CBTp are both efficacious treatments for paranoid ideations, but VR-CBTp may be somewhat more effective and more efficient than CBTp.

**Disclosure of Interest:**

None Declared

